# Boosting Evaporative Cooling Performance with Microporous Aerogel

**DOI:** 10.3390/mi14010219

**Published:** 2023-01-15

**Authors:** Huajie Tang, Chenyue Guo, Qihao Xu, Dongliang Zhao

**Affiliations:** 1 School of Energy and Environment, Southeast University, Nanjing 210096, China; 2 Engineering Research Center of Building Equipment, Energy, and Environment, Ministry of Education, Nanjing 210096, China

**Keywords:** evaporative cooling, radiative cooling, hydrogel layer, microporous aerogel layer

## Abstract

Hydrogel-based evaporative cooling with a low carbon footprint is regarded as a promising technology for thermal regulation. Yet, the efficiency of hydrogel regeneration at night generally mismatches with vapor evaporation during the day, resulting in a limited cooling time span, especially in arid regions. In this work, we propose an efficient approach to improve hydrogel cooling performance, especially the cooling time span, with a bilayer structure, which comprises a bottom hydrogel layer and an upper aerogel layer. The microporous aerogel layer can reduce the saturation vapor density at the hydrogel surface by employing daytime radiative cooling, together with increased convective heat transfer resistance by thermal insulation, thus boosting the duration of evaporative cooling. Specifically, the microstructure of porous aerogel for efficient radiative cooling and vapor transfer is synergistically optimized with a cooling performance model. Results reveal that the proposed structure with a 2-mm-thick SiO_2_ aerogel can reduce the temperature by 1.4 °C, meanwhile extending the evaporative cooling time span by 11 times compared to a single hydrogel layer.

## 1. Introduction

The global warming effect brings concern about the rising cooling load, which has accounted for ~15% of electricity use in the world [1]. As an alternative cooling approach, evaporative cooling draws abundant attention in a range of fields, including building cooling [2], semiconductor device cooling [3], food storage [4], as well as human-body thermal management [5,6]. Mimicking the sweat glands of mammals, evaporative coolers can efficiently dissipate parasitic heat by the vaporization of water, which releases heat at approximately 2400 J g^−1^. Mishra et al. developed a 3D-printed hydrogel actuator consisting of a poly-N-isopropylacrylamide (PNIPAm) body and a microporous (~200 μm) polyacrylamide (PAAm) dorsal layer. The hydrogel actuator can enhance the cooling rate by 600% over similar non-evaporating devices. Similarly, a thin layer of lithium- and bromine-enriched PAAm hydrogel was presented by Pu et al. [3]. They realized a temperature reduction of 17 °C and an efficiency improvement of 0.5% for commercial polycrystalline silicon solar cells under one sun condition. Yet, the efficiency of hydrogel regeneration at night generally mismatches with vapor evaporation during the day, resulting in a limited cooling time span, especially in arid regions. Hygroscopic salt can efficiently extend the cooling time span by assisting hydrogel regeneration [7]. Ji et al. proposed a self-restoring and environmentally adaptive hydrogel composed of PNIPAm and calcium alginate [2]. The hydrogel exhibits a sub-ambient cooling performance of ~13 °C and a hygroscopicity of ~1.71 g g^−1^. However, the combination of water molecules and salt ions can also increase the desorption resistance, and then decrease the daytime evaporative cooling power.

Another promising passive cooling technology is sky radiative cooling, which can efficiently dissipate heat to the cold space (~3 K) through the atmospheric window (8–13 μm) [8,9,10]. Since the first demonstration of radiative cooling below ambient temperature under direct sunlight at noon [11], a brunch of different types of radiative coolers have sprung up, such as photonic crystals [12,13], polymer metamaterial [14], paints [15,16], and porous structure [17,18]. Scalable and simple porous structure materials have greatly advanced the progress of this technology [19]. Hierarchically porous poly(vinylidene fluoride-co-hexafluoropropene) P(VdF-HFP) can realize a sub-ambient temperature reduction of ~6 °C and a cooling power of ~96 W m^−2^ under solar intensities of 890 and 750 W m^−2^, respectively [17]. To further enhance the cooling performance, Leroy et al. developed a 6-mm-thick polyethylene aerogel with a solar reflectivity of 92.2% and a low thermal conductivity of 0.028 W m^−1^ K^−1^ [20]. Benefiting from the thermal insulation, a temperature reduction of ~13 °C was monitored at noon (solar intensity ~1123 W m^−2^). Li et al. also proposed an all-ceramic, compressible and scalable nanofibrous aerogel for radiative cooling, yielding a power of ~133.1 W m^−2^ [21].

Recently, some researchers incorporated evaporative cooling with radiative cooling to improve passive cooling performance. Feng et al. developed a bilayer porous polymer for efficient passive building cooling, which comprises a hygroscopic Li-PAAm hydrogel and a hydrophobic porous P(VdF-HFP) top layer [22]. A remarkable sub-ambient temperature reduction of ~7 °C and an effective cooling power of ~150 W m^−2^ were obtained. Sun et al. designed a multiple-effect cooling fabric, consisting of a porous P(VdF-HFP) fiber and a cotton fabric loaded with CaCl_2_ [23]. At night, the underlying fabric preloaded with hygroscopic salt can capture the moisture in the air automatically. During the day, the evaporation and infrared radiation synergies, leading to a temperature reduction of 10.8 °C. By replacing the hundreds-microns-thick radiative cooling layer with a few-millimeter-thick cellulose acetate fibrous network, a tandem radiative/evaporative (TRE) cooling system was proposed by Li et al. [24]. They experimentally demonstrated a cooling power above ~300 W m^−2^ in different climate regions. These studies greatly highlighted the superimposed effect of the association of evaporating and daytime sky radiative cooling in temperature reduction. However, except for temperature reduction, the evaporating cooling time span is also an important cooling performance index, which has been rarely studied [25].

**Figure 1:**
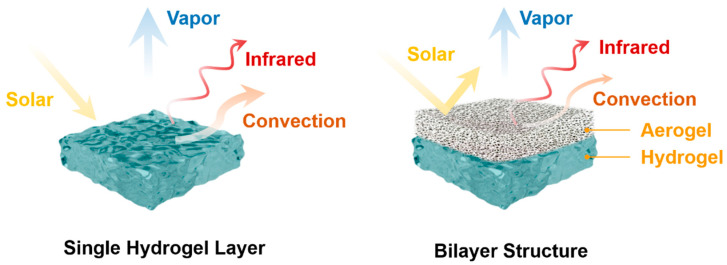
Schematic of working principles of a single hydrogel layer (**left**) and a hydrogel/aerogel bilayer structure (**right**).

In this work, we propose an efficient approach to boost evaporative cooling performance with a bilayer structure (BLS), which comprises a bottom hydrogel layer and an upper porous aerogel layer. As shown in Figure 1, the microporous aerogel can reflect solar irradiation strongly and isolate external convection heat transfer efficiently, increasing the temperature reduction. Taking advantage of the lower vapor density and the moisture transfer resistance of the aerogel, the cooling time span is greatly extended. Specifically, an optical model and a thermodynamics model were developed to simulate the cooling performance. The impacts of the detailed structure for the microporous aerogel were investigated, as well as the external weather conditions. Moreover, the cooling potential around China was further researched to discuss the applicability of the BLS strategy in the real-world. Our work deeply reveals the combining mechanism of evaporative cooling and radiative cooling.

## 2. Methodology

**Figure 2:**
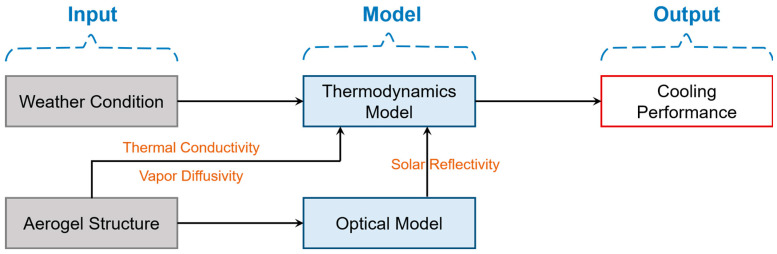
Schematic of the modeling process.

Herein, the improved cooling performance of the proposed bilayer strategy was systematically investigated with the perspective of the impacts of the intrinsic microstructure and external environmental conditions. Firstly, an optical model was developed to calculate the solar reflectivity of the aerogel layer. Subsequently, a thermodynamics model associated with evaporation and radiative cooling was proposed for cooling performance modeling. Together with the simulated solar reflectivity, the effective thermal conductivity and vapor diffusivity of the aerogel was input to build the physical model of the bilayer structure. The following parameters are the weather conditions, which indicate the different operating situations. Finally, the cooling performance was the output as shown in Figure 2.

### 2.1. Optical Model

The optical performance of the BLS is mainly determined by the upper layer aerogel. The reason of the optical dependence can be attributed to the super transparent appearance of the bottom layer hydrogel (with extremely low solar reflectivity). The homogeneous structure of the hydrogel layer is composed of a mass of water molecules (~98 wt%) and a few macromolecular chains [22,25]. Meanwhile, very limited incident irradiation (less than 5%) can go through the microporous aerogel with a thickness above ~200 μm [26]. Given all these, we consider the reflectivity of the aerogel layer as that of the BLS here. For a porous medium with low dielectric loss, the micro- or/and nanostructure can strongly scatter the incident electromagnetic wave with the corresponding size x=πD/λ, resulting in a super white appearance. The scattering efficiency $Q_{sca}$ of a single particle or fiber can be expressed as [27]:

(1) $$Q_{sca}=\frac{2}{x^{2}}\overset{\infty }{\underset{n=1}{\sum }}\left(2n+1\right)\left({\left|a_{n}\right|}^{2}+{\left|b_{n}\right|}^{2}\right)$$

where x=πD/λ is the size parameter. D, λ refers to the fiber diameter and incident wavelength, respectively. As well, the Mie coefficients of $a_{n}$, $b_{n}$ can be given from the relative refractive index and magnetic permeability [15].

The direction for the scattering behavior can be described by the asymmetry factor g:

(2) $$g=\frac{1}{4\pi }\int_{0}^{4\pi }\phi \left(\Theta \right)\cos\Theta d\Omega$$

where ϕ(Θ) is the scattering phase function.

Meanwhile, the scattering coefficient σ of the fiber coma per unit length along the incident direction can be ascribed as the join effect of the monomers.

(3) $$\sigma =NC_{sca}=NQ_{sca}G$$

where N is the number of fibers per unit volume, $C_{sca}$ and G are the scattering cross-section and the projected area, respectively. The scattering phase function Φ(Θ) and asymmetry factor g of the coma are the same as that of a single fiber.

In this way, the radiation transfer process in the porous medium can be modified as:

(4) $$\frac{dI_{\lambda }\left(s\right)}{ds}=-\kappa_{\lambda }I_{\lambda }\left(s\right)-\sigma_{\lambda }I_{\lambda }\left(s\right)+\kappa_{\lambda }I_{b\lambda }\left(s\right)+\frac{\sigma_{\lambda }}{4\pi }\int_{0}^{4\pi }I_{\lambda }\left(s,\vec{\Omega^{\prime }}\right)\phi_{\lambda }\left(\vec{\Omega^{\prime }},\vec{\Omega }\right)d\Omega^{\prime }$$

where $I_{\lambda }\left(s\right)$ is the spectral radiation intensity in the direction of $\vec{\Omega }$ (solid angel Ω) along path s, $\kappa_{\lambda }$ is the absorption coefficient. In this work, we use SiO_2_ aerogel, the absorption index of which in the solar range is much smaller than the refractive index, so it is considered as a non-absorbent medium. We solved the radiation transfer equation by the Monte Carlo Ray Tracing method.

### 2.2. Thermodynamics Model

**Figure 3:**
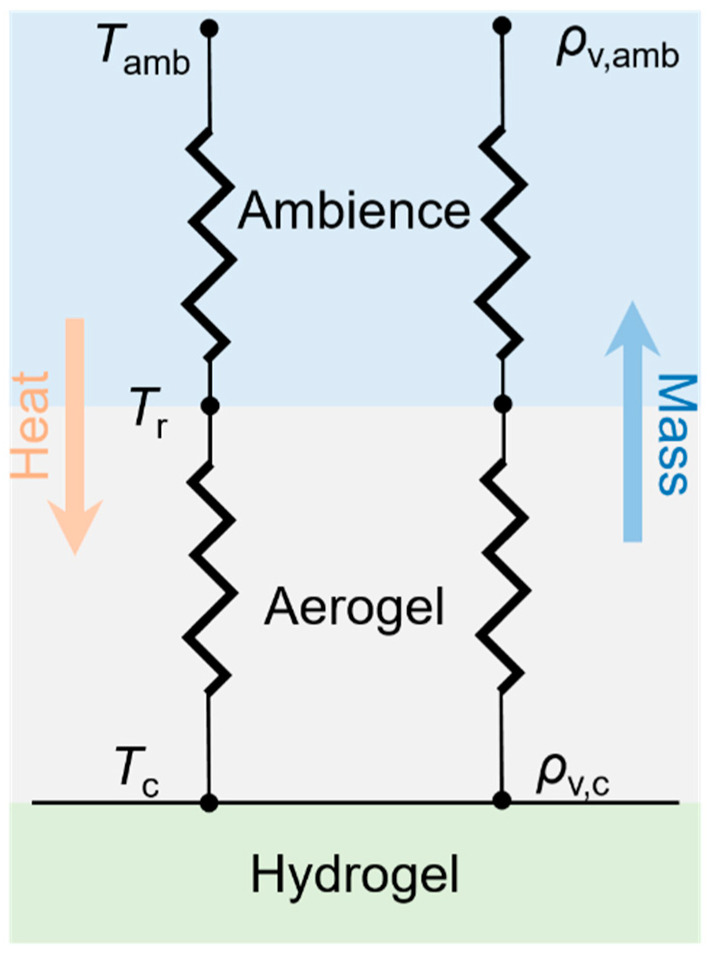
Schematic of heat and mass transfer processes for the bilayer structure.

According to the energy conservation law, a thermodynamics model for the bilayer structure was established here. As illustrated in Figure 3, there exist two different transfer processes, namely inward heat flow and outward mass flow. Therefore, passive cooling power $P_{net}$ is composed of radiative cooling power $P_{rad}\left(T_{r}\right)$ and evaporative cooling power $P_{eva}\left(T_{c}\right)$.

(5) $$P_{net}=P_{rad}\left(T_{r}\right)+P_{eva}\left(T_{c}\right)$$

where $T_{r}$ and $T_{c}$ are the temperatures of the aerogel radiative cooler and the hydrogel evaporator, respectively.

Specifically, radiative cooling power provided by the aerogel layer can be given by:

(6) $$P_{rad}\left(T_{r}\right)=P_{r}\left(T_{r}\right)-P_{atm}\left(T_{atm}\right)-P_{sun}-P_{cond+conv}$$

where $P_{r}\left(T_{r}\right)$ and $P_{atm}\left(T_{atm}\right)$ are the emitted IR radiation and absorbed atmospheric radiation respectively, which can be defined as follows:

(7) $$P_{r}\left(T_{r}\right)=2\pi \int_{0}^{\frac{\pi }{2}}d\theta sin\theta cos\theta \int_{2.5\mu m}^{25\mu m}d\lambda I_{B}\left(T_{r},\lambda \right)\sigma_{r}\left(\theta ,\lambda \right)$$

(8) $$P_{atm}\left(T_{atm}\right)=2\pi \int_{0}^{\frac{\pi }{2}}d\theta sin\theta cos\theta \int_{2.5\mu m}^{25\mu m}d\lambda I_{B}\left(T_{atm},\lambda \right)\sigma_{r}\left(\theta ,\lambda \right)\sigma_{atm}\left(\theta ,\lambda \right)$$

where $\sigma_{r}\left(\theta ,\lambda \right)$ and $\sigma_{atm}\left(\theta ,\lambda \right)$ are the directional spectral emissivity of the aerogel layer and atmosphere. According to the Kirchhoff’s law, the emissivity equals to the absorptivity. As well, $I_{B}\left(T,\lambda \right)$ is the spectral radiation of black body with temperature T.

For daytime cooling, the solar absorption portion $P_{sun}$ should be carefully considered.

(9) $$P_{sun}=\int_{0.25\mu m}^{2.5\mu m}d\lambda I_{sun}\left(\lambda \right)\alpha \left(\lambda ,\theta_{sun}\right)cos\left(\theta_{sun}\right)$$

where, $I_{sun}\left(\lambda \right)$ is the spectral solar irradiation and $\theta_{sun}$ is the incident angle of sunlight. $\alpha \left(\lambda ,\theta_{sun}\right)$ is the spectral angular absorptivity of the aerogel in the solar region.

The non-radiation heat transfer flux $P_{cond+conv}$ includes conduction and convection heat exchange between the upper surface and the surrounding media.

(10) $$P_{cond+conv}=h_{c}\left(T_{amb}-T_{r}\right)$$

where $h_{c}$ is the non-radiative heat transfer coefficient, which can be described as a liner function of wind speed *v* [28].

(11) $$h_{c}=8.3+2.5v$$

On the other hand, the passive cooling profit induced by evaporation depends on the difference in the vapor mass density between the hydrogel surface $\rho_{v,c}$ and that in the ambient $\rho_{v,amb}$. It can be calculated by:

(12) $$P_{eva}\left(T_{c}\right)=h_{fg}\frac{\rho_{v,c}-\rho_{v,amb}}{R_{aer}}$$

where $h_{fg}$ is the enthalpy of vaporization of water in the hydrogel at the temperature T.

(13) $$h_{fg}=0.001\left(2500+1.84\left(T_{s}-273.15\right)\right)$$

The overall mass transfer resistance $R_{aer}$ includes the diffusion resistance in the aerogel $t_{aer}/D_{aer}$ and the external mass convection resistance $1/g_{ext}$.

(14) $$R_{aer}=\frac{t_{aer}}{D_{aer}}+\frac{1}{g_{ext}}$$

Generally, a common measurement method for vapor diffusivity $D_{aer}$ is the wet-cup method following ASTME96. For simplification, a formula for the effective diffusion coefficient of porous media based on the Bruggeman equation [29] is adopted here, which adjusts the binary diffusion $D_{0}$ with porosity f.

(15) $$D_{aer}=D_{0}f^{1.5}$$

The cooling temperature $T_{c}$, i.e., the temperature of the hydrogel can be obtained at the quasi-steady state, when the evaporating heat absorption rate equals to the radiative cooling power with no enthalpy change for the hydrogel layer.

(16) $$T_{c}=\frac{P_{rad}\left(T_{r}\right)}{\frac{k_{aer}}{t_{aer}}}+T_{r}$$

where $k_{aer}$ is the effective thermal conductivity of aerogel, which incorporates heat conduction, convection, and radiation. According to Hrubesh et al. [30], the conductivity $k_{aer}$ can be expressed as follow:

(17) $$k_{aer}=k_{s}+k_{g}+k_{r}$$

where $k_{s}$, $k_{g}$ and $k_{r}$ are the thermal conductivities of the solid phase, gaseous phase, and radiation, respectively. The calculation details can be found in Supporting Information.

Besides temperature reduction $\Delta T=T_{atm}-T_{c}$, another important cooling performance indicator, cooling time $\tau_{c}$ is determined as follows:

(18) $$\tau_{c}\frac{\rho_{v,c}-\rho_{v,amb}}{R_{aer}}=t_{h}\rho_{h}\omega$$

where $t_{h}$ and $\rho_{h}$ repent the thickness and the density of the hydrogel layer. ω is the initial water mass fraction of the hydrogel. In the work, a 5-mm-thick acrylamide hydrogel [25] was chosen.

## 3. Results

### 3.1. Model Validation

**Figure 4:**
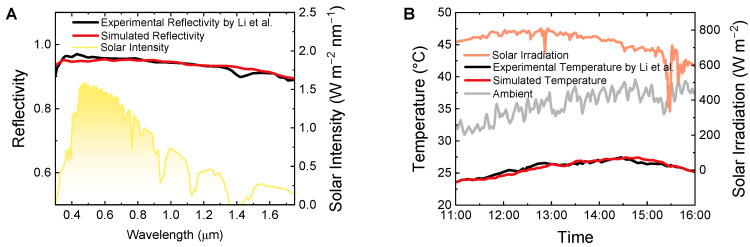
Validation of the optical model (**A**) and the thermodynamics model (**B**). The experimental data were obtained from Li et al. [24].

In this section, the established optical model and thermodynamics model were validated based on the recent work by Li et al. [24]. They developed a tandem radiative/evaporative (TRE) cooling system to demonstrate the superimposed cooling performance. It should be stressed that the originality of this work lies on the thoroughly researched effect mechanism of the external weather and BLS structure on improved cooling performance, rather than proposing a specific cooler as usual. The referenced TRE cooler consists of the cellulose acetate fibrous network and poly(vinyl alcohol)–CaCl_2_ hydrogel materials, which possesses a high solar reflectivity of 0.95 (Figure 4A). With a solar irradiation of ~700 W m^−2^ and an ambient temperature of ~35 °C, a passive temperature reduction of ~10 °C was monitored in a clear day (Figure 4B). We implemented the solar reflectivity simulation for the TRE cooler with the reflective index of cellulose (Figure S1) and the structure parameter of the cellulose network (e.g., a diameter size distribution of around 0.5 to 0.85 μm). The simulated cooling temperature was obtained by iterative calculation with the meteorological parameters. It can be seen from Figure 4 that the simulated curves fit well with the experimental data. To quantify the data deviation, we adopted the root mean square error (RMSE) here as the indicator ($\mathrm{RMSD}=\sqrt{\frac{\sum_{i=1}^{n}{\left(\frac{V_{sim,i}-V_{exp,i}}{V_{exp,i}}\right)}^{2}}{n}}$). The RMSE for the simulated solar reflectivity and temperature are 0.012 and 0.013 respectively, accounting for extremely low differences. Such high precision of the simulation demonstrates the validity of the proposed models.

### 3.2. Aerogel Properties

The passive cooling performance of the BLS relies on the detailed structure. To investigate the effect of upper aerogel layer, we researched the optical property and heat mass transfer property systematically.

**Figure 5:**
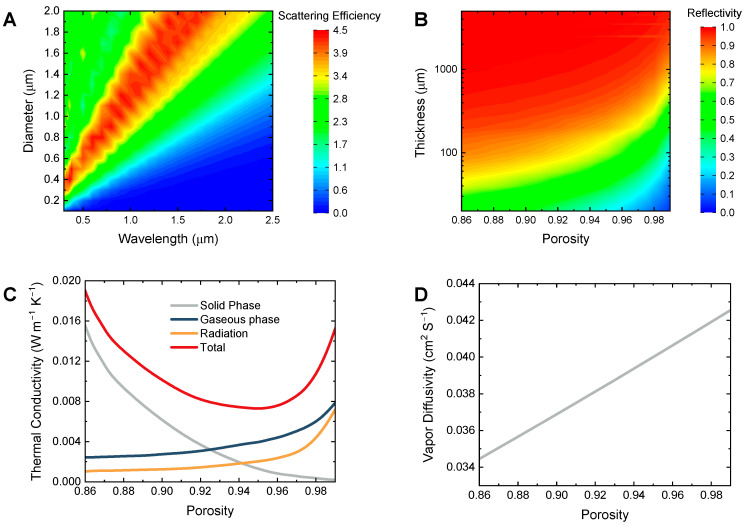
Parameters of aerogel layer response to its microstructure. Simulated scattering efficiency (**A**) and solar reflectivity (**B**) based on the optical model. Calculated thermal conductivity (**C**) and vapor diffusivity (**D**).

Figure 5A shows the scattering efficiency of different fiber diameter D in different wavelengths. As mentioned above, the peak of the Mie scattering efficiency $Q_{sca}$ occurs when the size parameter x approximately equals 1. So that the scattering peak broadens with the increase of diameter, meanwhile shifting to the near-infrared (NIR) band. For example, when the diameter is 0.4 μm, the irradiation can be rejected in the visible band, but not in the NIR band. To efficiently reflect the irradiation in the whole solar region, the range where the scattering peak locates is supposed to surpass that of the solar intensity peak. Considering the solar spectrum of AM 1.5, the irradiation intensity of which during 0.4–1.1 μm wavelength accounts for ~75% of solar energy, we chose the diameter of 0.8 μm for further study. Figure 5B presents the solar reflectivity of the aerogel with a diameter of 0.8 μm for different thicknesses and porosity. It can be found that the solar reflection effect becomes progressively significant with the thickness increasing and porosity decreasing, especially when the thickness is less than 100 μm. However, when the thickness is larger than 1000 μm, the aerogel shows a universal high reflectivity above 0.9. The detailed reason can be explained as follow. Generally, the multiple scattering effects can be improved with the increase of the effective path length of the incident wave, which can be represented by the aerogel thickness and the porosity. So, when the path length is long enough, the increase of the reflectivity becomes less. For the aerogel layer with a thickness above 1000 μm and a diameter of 0.8 μm, the BLS can maintain a high reflectivity.

The heat and mass transfer properties were studied based on the thermodynamics model. As illustrated in Figure 5C, the gaseous phase conductivity and the radiation one increase with the porosity, but the solid phase conductivity presents a decreasing trend. In synergy, the total thermal conductivity decreases firstly and then increases with the porosity increasing, the turning point of which is 0.95. As for vapor transfer, it can be roughly described as a linear relation with the porosity, as shown in Figure 5D. When the porosity increases from 0.86 to 0.99, the vapor diffusivity can be enhanced from 0.0345 to 0.0426 cm^2^ S^−1^. It can be easily understood that a high porosity characterizes a low diffusivity resistance.

### 3.3. The Effect of Aerogel Structure Parameters on Cooling Performance

**Figure 6:**
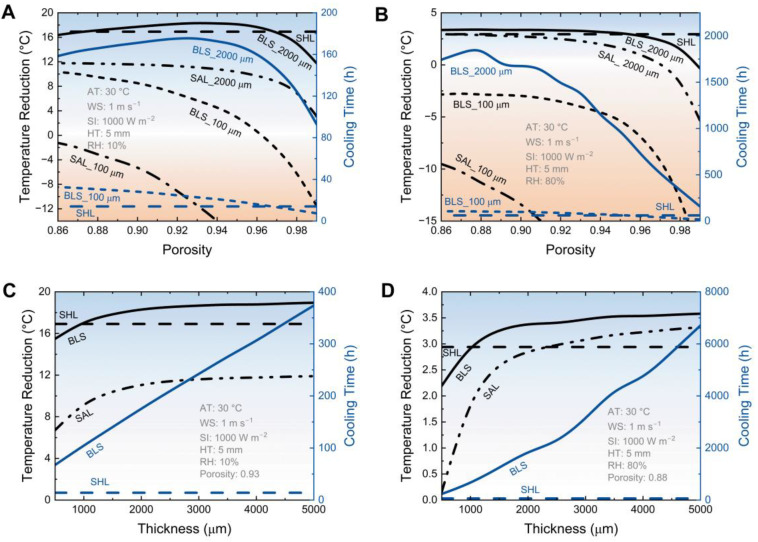
The effect of aerogel structures on cooling performance. Simulated temperature reduction and cooling time span with different porosity at dry (**A**) and humid (**B**) conditions. Simulated temperature reduction and cooling time span with different thicknesses at dry (**C**) and humid (**D**) conditions.

To further investigate the effect of the aerogel, the cooling performance of the BLS with different aerogel porosity and thickness was modeled (Figure 6). As a typical scenario, the assumed ambient temperature (AT) is 30 °C, the solar irradiation (SI) is 1000 W m^−2^, the wind speed (WS) is 1 m s^−1^, and the thickness of the hydrogel layer (HT) is 5 mm. When in the dry region with relative humidity (RH) of 10%, the temperature reduction and cooling time span increase firstly then decrease with the increase of porosity for the BLS with a 2000-μm-thick-aerogel. The maximum temperature reduction at the porosity of 0.93 is 18.3 °C, which is higher than that of the single hydrogel layer (SHL) (16.9 °C), as well as the single aerogel layer (SAL) (11.1 °C). Simultaneously, the maximum cooling time (175.7 h) is even more than twelve times that of SHL (14.0 h). The targeted aerogel porosity of 0.93 is determined by the cooperation effect of the solar reflection, thermal insulation, and vapor diffusivity. However, when the thickness of the aerogel layer is 100 μm, the temperature reduction and cooling time span of the BLS decrease with the porosity. Because the solar reflectivity of the thin aerogel fluctuates widely with the porosity, the solar absorption dominates the heat gain. Due to the low solar reflectivity, the temperature reduction is 6.6 °C less than that of SHL, but the cooling time span can still be doubled for the BLS with the aerogel porosity of 0.86.

In humid regions (RH 80%), the evolution trends for the temperature reduction and cooling time span of the BLS are similar to that of the dry regions, respectively (Figure 6B). But the desired porosity is 0.88, rather than 0.93. The reason behind that is that thermal insulation makes a bigger difference than the radiative cooling and evaporation in the humid region, leading to the turning point shifting to low porosity. While the maximum temperature reduction of the BLS with a 2000-μm-thick-aerogel is just 0.5 °C higher than that of SHL, the cooling time is as long as 1880.0 h, almost 30 times that of SHL. The greatly extended cooling time span efficiently boosts the cooling performance.

Following the porosity study, the impact of aerogel thickness on the cooling performance of the BLS is discussed. Figure 6C shows the cooling performance of the BLS with different aerogel thicknesses in a dry environment. It can be found that both the temperature reduction and cooling time of the BLS increase with the aerogel thickness. The difference is that the growth rate of the former gradually decreases with the increase of thickness, but the latter shows a linear increasing trend. When the aerogel thickness excesses ~1000 μm, the temperature of the BLS is cooled below that of SHL. As well, when the thickness excesses ~2000 μm, the temperature of the BLS changes slowly. The enhancement of aerogel thickness for the cooling performance is more significant in a humid region. As shown in Figure 6D, the temperature reduction of the BLS with a 5000-μm-thick-aerogel increases by 21.7% over that of SHL, much larger than the increase in the dry region (12.0%). Meanwhile, the cooling time span increases by more than one hundred times due to the extremely low vapor density difference in a humid region. This superior cooling performance demonstrates the significant improvement with the BLS strategy.

### 3.4. The Effect of Weather Conditions on Cooling Performance

**Figure 7:**
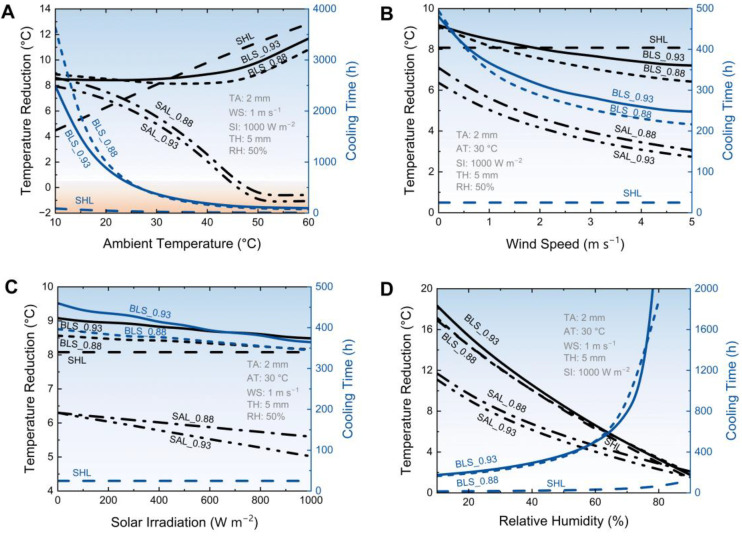
The effect of weather conditions on cooling performance. Simulated temperature reduction and cooling time span with different ambient temperatures (**A**), wind speeds (**B**), solar irradiation (**C**), and relative humidity (**D**).

In addition to the microstructure of the aerogel, the external weather conditions also deeply influence the cooling performance of the BLS. Figure 7A shows the responsibility of the ambient temperature. It can be found that the temperature reduction of the BLS reveals a tendency of decreasing first and then increasing, because of the opposite slope for the temperature reduction curves of SHL and SAL. While the cooling time span of the BLS monotonically decreases with the ambient temperature. More interestingly, the BLS with a higher aerogel porosity (0.93) shows superior temperature reduction when the ambient temperature is higher than ~24 °C, but the one with a smaller porosity (0.88) is the better cooler when the environment is temperate. The applicability in the different ambience for the BLS with different aerogel porosities is caused by the different evaporating rates. Although in a hot environment (>33 °C), the temperature of the BLS can be 1 °C higher than that of SHL due to the evaporating resistance, the cooling time span can increase by 20 times.

Figure 7B shows that cooling performance attenuates with the increase of the wind speed. The increased wind speed enhances the conduction and convection heat exchange with the environment, so that the temperature reduction and cooling time are decreased simultaneously. It can be found that the BLS with a higher aerogel porosity maintains larger temperature reduction and longer cooling time, because of the lower thermal conductivity. When the wind speed is around 2 m s^−1^, accounting for a non-radiation heat exchange coefficient of 13.3 W m^−2^ K^−1^, the temperature reduction of the BLS is 8.0 °C, and the cooling time is 312.1 h.

Figure 7C shows that the cooling performance of the BLS weakens with the increase of the solar irradiation gradually. For the BLS with an aerogel porosity of 0.93, as the intensity increases from 0 to 1000 W m^−2^, the temperature reduction decreases by 0.6 °C and the cooling time reduces 95.5 h, corresponding to an attenuation rate of 6.5% and 20.7% respectively. For the BLS with an aerogel porosity of 0.88, the temperature reduction and the cooling time reduce 0.4 °C and 51.8 h respectively, and the corresponding attenuation rate is 4.8% and 13.1%. The distance between the BLSs with different aerogel porosity decreases with the increase of solar irradiation.

Figure 7D shows the cooling performance response to the relative humidity. With the increase of the humidity, the temperature reduction of the BLS decreases but the cooling time span increases. Specifically, when the humidity is less than 70%, the BLS with an aerogel porosity of 0.93 can provide a larger temperature reduction. As well, it is in another case when the humidity is larger than 70%. As for the cooling time, when the humidity is less than 60% or larger than 75%, the BLS with the aerogel porosity of 0.93 presents a stronger persistence. In the narrow humidity range between 60–75%, the BLS with the aerogel porosity of 0.88 seems better. These intriguing results show the applicability of the different kinds of BLS. Notably, when the humidity is larger than 80%, the temperature of the BLS is below the dew-point (28.7 °C), and it starts to the condenser, rather than evaporate.

## 4. Discussion

**Figure 8:**
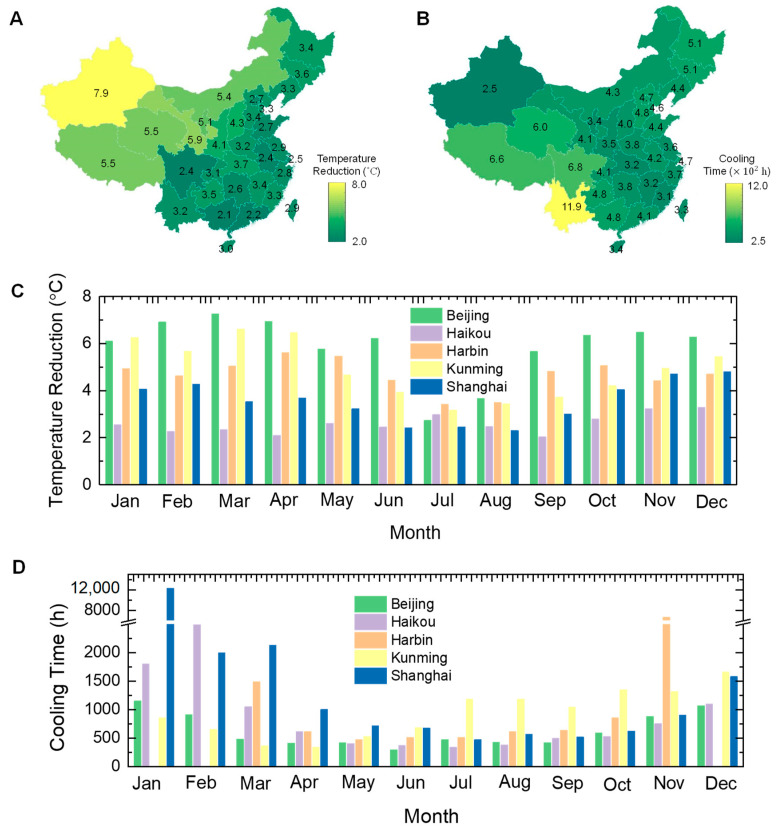
Cooling potential of the proposed bilayer structure in China. Simulated mean temperature reduction (**A**) and cooling time (**B**) in July. Simulated monthly temperature reduction (**C**) and cooling time (**D**) for different climate regions.

The weather sensibility analysis has demonstrated the BLS strategy can efficiently boost the cooling performance of the SHL in a set of complex environments, especially the working persistence. To further study the real application performance, the cool potential in China was investigated. Figure 8A presents the temperature reduction of the BLS in the provincial capital cities in July. The simulation was based on the BLS, consisting of a 2-mm-thick aerogel layer (porosity 0.93 and diameter 0.8 μm) and a 5-mm-thick hydrogel layer. The daytime means meteorological parameters in July were obtained from Chinese Standard Weather Data (CSWD) (Table S1). It can be found that the cities in northwestern China contain lager temperature reductions, and the coastal cities including the humid Sichuan basin possess the poor cooling ability. This uneven distribution may be caused by the difference in the humidity. The maximum temperature reduction in Urumchi city of Xinjiang Uygur Autonomous Region can be 7.9 °C, but the minimum one in Nanning city of Guangxi Zhuang Autonomous Region is just 2.1 °C.

Figure 8B shows the cooling time span of the BLS in the provincial capital cities in July. Unlike the distribution pattern of the temperature reduction, the cities located in the southwest and the northeast of China have longer cooling times. Because the cooling time is not only associated with the relative humidity, but also the ambient temperature. So that the BLS can present excellent persistence in the high altitudes and latitudes regions. The maximum cooling time for the thin BLS in Kunming city of Yunnan province can be as long as 1190 h.

Moreover, we also simulated the monthly temperature reduction and cooling time for different climate regions. The detailed meteorological parameters for the representative cities was displayed in Tables S2–S6. Figure 8C shows that the temperature reductions decrease first and then increase with the month, except in the hot region (Haikou), where the temperature reduction fluctuates between 2–3 °C. The valley of the temperature reductions for different climate regions are all in July, and the distinction is the month with maximum temperature reduction. For example, the maximum temperature reduction for the severe cold region (Harbin) is in December, but that for the temperate region (Kunming) is in March. Compared with the SHL (Figure S2), the BLS shows superiority in most of the months for different regions, except for the hot summer and cold winter region (Shanghai). The underperformance of the BLS in Shanghai can be attributed to the coupled effect of poor radiative cooling performance and limited evaporating due to year-round high ambient humidity. While the enhanced temperature reduction in other regions can reach above 4 °C, and the weakened temperature reduction is just less than 1 °C. It should be stressed that the main improvement for the BLS is the greatly extended cooling time span, which can be boosted by one or more orders of magnitude.

Figure 8D shows the monthly cooling time for different regions. Although the cooling time for different regions all reveals the trend for decreasing first and then increasing, the exact month when the values reach the valley or the peak are not the same. In the hot summer and cold winter region (Shanghai), the maximum cooling time is in January and the minimum one is in July. But in the severe cold region (Harbin), the maximum cooling time is in November and the minimum one is in May. It also should be noticed that, in the severe cold region, the temperature of the BLS has reduced below the dew-point temperature in the winter, and stopped evaporating.

## 5. Conclusions

In this work, a strategy for boosting hydrogel-based evaporative cooling performance with microporous aerogel was proposed. The improved cooling performance of the bilayer structure was simulated based on a thermodynamics model, associated with an optical model. With a 2-mm-thick SiO_2_ porous aerogel (porosity 0.93 and diameter 0.8 μm), the temperature reduction and cooling time span for a 5-mm-thick hydrogel are enhanced by ~1.4 °C and more than 11 times respectively, even under a strong solar intensity of 1000 W m^−2^. The cooling performance impacts of the aerogel structure and external weather conditions were thoroughly researched to analyze the affecting mechanism of the bilayer structure. Moreover, the regional and monthly cooling potential around China was systematically studied. In the hottest July, the maximum temperature reduction can be 7.9 °C and the cooling time span can reach as long as 1190 h. The results well demonstrated the improved evaporative cooling performance, especially the total working time.

## Data Availability

Not applicable.

## Supplementary Materials

The following supporting information can be downloaded at: https://www.mdpi.com/article/10.3390/mi14010219/s1, Figure S1: Refractive index of cellulose; Figure S2: Simulated improved monthly temperature reduction in different representative cities; Table S1: Mean daytime meteorological parameters in July; Table S2: Monthly mean daytime meteorological parameters in Beijing (cold region); Table S3: Monthly mean daytime meteorological parameters in Harbin (severe cold region); Table S4: Monthly mean daytime meteorological parameters in Shanghai (hot summer and cold winter region); Table S5: Monthly mean daytime meteorological parameters in Kunming (temperate region); Table S6: Monthly mean daytime meteorological parameters in Haikou (hot region).

## Glossary

**Nomenclature**	
$a_{n},\;b_{n}$	Mie coefficients
D	diameter of the fiber, m
f	porosity
g	asymmetry factor
$h_{c}$	non-radiative heat coefficient, W m^−2^ K^−1^
$h_{fg}$	enthalpy of vapor, kJ kg^−1^
$I_{B}\left(T,\lambda \right)$	the spectral radiance of a black body, W
$I_{sun}\left(\lambda \right)$	solar illumination, W m^−2^
k	thermal conductivity, W m^−1^ K^−1^
$P_{atm}$	absorbed IR radiation, W m^−2^
$P_{cond+conv}$	non-radiation heat transfer, W m^−2^
$P_{eva}$	evaporative cooling power, W m^−2^
$P_{net}$	net passive cooling power, W m^−2^
$P_{r}$	emitted IR radiation, W m^−2^
$P_{rad}$	radiative cooling power, W m^−2^
$P_{sun}$	solar intensity absorbed by radiative paints, W m^−2^
$Q_{sca}$	scattering efficiency
$R_{aer}$	mass transfer resistance, s m^−1^
$T_{c}$	cooling temperature, K
t	thickness, m
x	size parameter
**Greek symbols**	
α	spectral and angular solar absorptivity
λ	length of incident wave, m
$\rho_{h}$	density of the hydrogel layer, kg m^−3^
$\rho_{v}$	vapor mass density, g kg^−1^
σ	scattering coefficient of the fiber coma
$\sigma_{r}$	spectral and angular emissivity in the infrared region
τ	cooling time, h
Ω	solid angel for scattering
ω	water mass fraction of the hydrogel
**Superscripts and subscripts**	
aer	aerogel
amb	ambient
atm	atmospheric
ext	external
g	gaseous phase
h	hydrogel
r	radiation
s	solid phase
