# Supplementary material for: Boosting Evaporative Cooling Performance with Microporous Aerogel
Source: Micromachines (Basel). 2023 Jan 15;14(1):219. doi: 10.3390/mi14010219 (PMC9862351; doi:10.3390/mi14010219)
Supplement: Supplementary file 1 [file micromachines-14-00219-s001.zip › micromachines-2159815-supplementary.pdf]

micromachines

MDPI

Supplementary Materials: Boosting Evaporative Cooling Performance with Microporous Aerogel

Huajie Tang [1], Chenyue Guo [1], Qihao Xu [1] and Dongliang Zhao [1,2,*]

[1] School of Energy and Environment, Southeast University, Nanjing 210096, China
[2] Engineering Research Center of Building Equipment, Energy, and Environment, Ministry of Education, China, Nanjing 210096, China
* Correspondence: author: dongliang_zhao@seu.edu.cn

TABLE OF CONTENTS

Effective thermal conductivity of aerogel
Refractive index of cellulose (Figure S1)
Simulated improved monthly temperature reduction in different representative cities (Figure S2)
Meteorological parameter (Tables S1–S6)

Effective Thermal Conductivity of Aerogel

The effective thermal conductivity of aerogel k_{aer} is composed of solid conductivity k_s, gaseous conductivity k_g, and radiation conductivity k_r. Generally, the heat conduction through the solid structure of the aerogel can be given by:

$$k_s = k_0 \frac{d_s v_s}{d_0 v_0} \tag{S1}$$

Where d_s and d_0 are the density of the aerogel and the solid from which it is made, v_s and v_0 are the corresponding longitudinal sound velocity, and k_0 represents the thermal conductivity of the solid.

The d_s can also be represent by the porosity f:

$$f = 1 - \frac{d_s}{d_0} \tag{S2}$$

The gaseous conduction can be expressed as:

$$k_g = \frac{k_{g0} f}{1 + \alpha K_n} \tag{S3}$$

Where k_{g0} is the thermal conductivity of the gas in the pore, K_n is the Knudsen number, and the constant α for air is 2. It should be noted that the conduction gas is wet air with moisture, instead of dry air. However, due to the low volume fraction of water vapor in the wet air, we used the thermal conductivity of dry air for the calculation of wet air.

For radiation conductivity, it can be given as follow:

$$k_r = \frac{16 n^2 \sigma T_r^3 d_0}{3 d_s K_0} \tag{S4}$$

Where σ is the Stephan-Boltzmann constant, n is the refractive index (~1 for aerogels), T_r is the mean temperature within the material, and K_0 is the extinction coefficient for the solid.

With the sum of Equations (S1), (S3), and (S4), the effective thermal conductivity of aerogel k_{aer} can be expressed as a function of porosity f:

$$k_{aer} = k_0 \frac{v_s(1-f)}{v_0} + \frac{k_{g0} f}{1+\alpha K_n} + \frac{16 n^2 \sigma T_r^3}{3K_0(1-f)} \tag{S5}$$

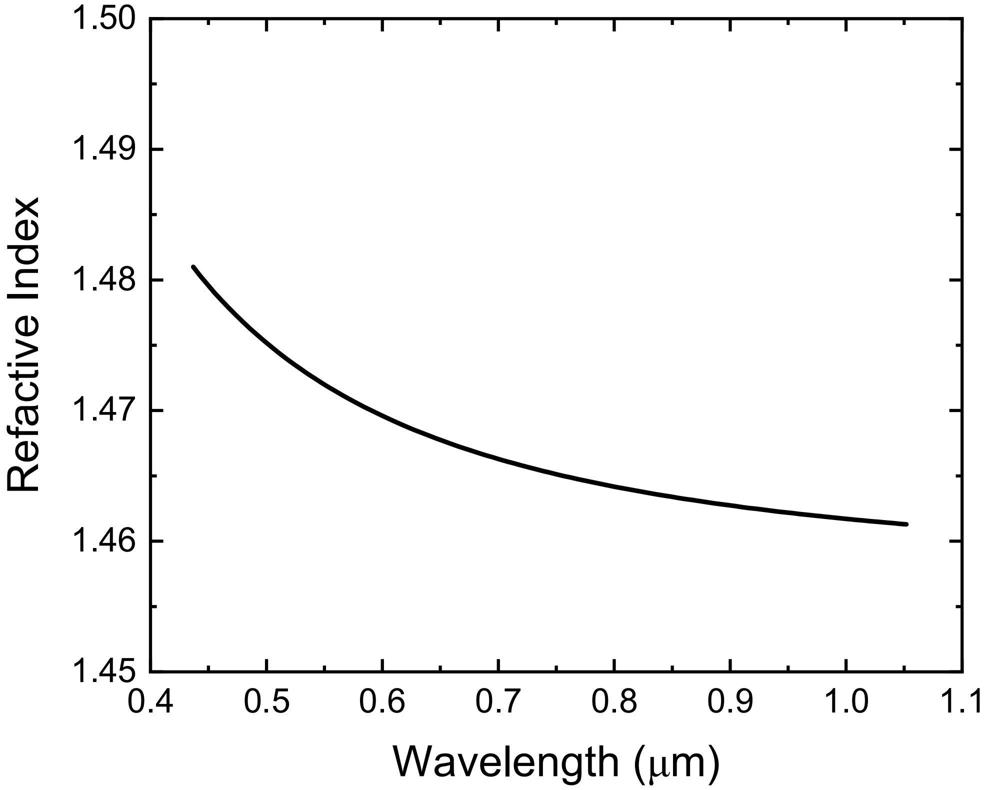

Figure S1. Refractive index of cellulose.

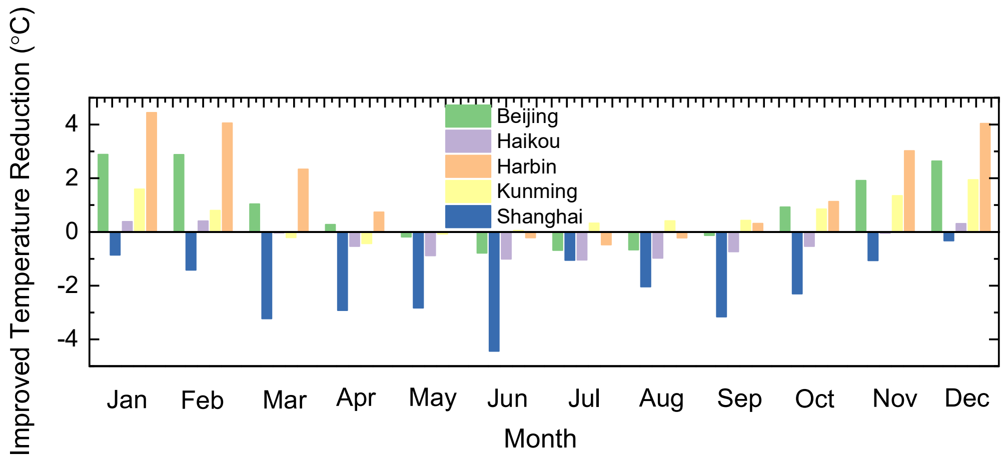

Figure S2. Simulated improved monthly temperature reduction in different representative cities.

Meteorological parameter

Table S1. Mean daytime meteorological parameters in July.

Province	City	Ambient Temperature (˚C)	Relative Humidity	Solar Irradiation (W m^{-2})	Wind Speed (m s^{-1})	Cloudage
Tianjin	Tianjin	27.0	72.2	312.6	1.8	4.5
Anhui	Hefei	28.8	77.2	0.8	296.7	3.0
Beijing	Beijing	27.4	75.9	328.1	2.0	4.5
Fujian	Fuzhou	30.3	70.7	372.2	3.6	4.6
Gansu	Lanzhou	23.8	55.9	401.4	1.3	4.6
Guangdong	Guangzhou	29.7	79.0	277.2	1.8	4.7
Guangxi	Nanning	29.0	80.6	323.5	1.5	4.5
Guizhou	Guiyang	25.1	70.4	337.8	3.1	4.5
Hainan	Haikou	30.5	73.4	417.1	2.4	4.5
Hebei	Shijiazhuang	27.1	72.5	298.2	1.6	4.3
Henan	Zhenzhou	28.5	71.9	361.6	2.6	4.5
Heilongjiang	Harbin	23.8	70.5	319.3	3.6	4.6
Hubei	Wuhan	31.0	69.5	376.8	1.7	4.1
Hunan	Changsha	29.8	75.9	364.0	2.0	4.5
Jilin	Changchun	24.1	69.8	297.1	3.3	4.3
Jiangsu	Nanjing	30.0	73.9	320.0	2.7	4.5
Jiangxi	Nanchang	30.4	71.0	416.1	2.8	4.4
Liaoning	Shenyang	26.6	71.8	310.4	3.9	4.4
Inner Mongolia	Hohhot	23.7	58.6	381.9	1.4	4.7
Ningxia	Yinchuan	25.8	59.3	444.2	2.5	4.5
Qinhai	Xining	19.4	58.2	446.2	1.6	4.4
Shandong	Jinan	27.7	75.5	276.5	2.6	4.8
Shanxi	Taiyuan	25.6	64.8	349.2	2.4	4.6
Shannxi	Xi'an	28.2	66.9	335.3	2.1	4.5
Shanghai	Shanghai	28.3	77.8	336.3	3.4	4.4
Sichuan	Chengdu	26.9	80.2	278.3	1.3	4.2
Taiwan	Taipei	30.7	74.0	395.3	3.0	4.5
Tibet	Lhasa	17.5	58.0	480.6	1.5	4.8
Hongkong	Hongkong	29.7	78.8	414.3	3.2	5.3
Xinjiang	Urumchi	24.9	41.0	405.1	2.4	4.5
Yunnan	Kunming	21.0	76.6	310.8	1.6	4.5
Zhejiang	Hangzhou	29.4	74.3	347.8	2.5	4.7
Chongqing	Chongqing	29.1	73.9	328.2	1.6	4.4

Table S2. Monthly mean daytime meteorological parameters in Beijing (cold region).

Month	Ambient Temperature (˚C)	Relative Humidity	Solar Irradiation (W m^{-2})	Wind Speed (m s^{-1})	Cloudage
Jan	-2.2	34.8	247.8	2.9	4.6
Feb	0.6	30.8	314.5	2.5	4.5
Mar	9.2	30.3	355.0	3.1	4.5
Apr	16.4	42.0	394.4	2.9	4.4
May	20.9	53.8	373.9	2.7	4.4
Jun	25.8	52.0	349.2	2.7	4.4
Jul	27.4	75.9	328.1	2.0	4.5
Aug	26.8	69.8	343.8	2.2	4.4
Sep	22.9	56.4	309.1	2.3	4.4
Oct	15.2	49.5	295.3	2.2	4.6
Nov	8.4	46.4	250.0	2.1	4.6
Dec	1.5	39.7	218.1	2.7	4.6

Table S3. Monthly mean daytime meteorological parameters in Harbin (severe cold region).

Month	Ambient Temperature (˚C)	Relative Humidity	Solar Irradiation (W m^{-2})	Wind Speed (m s^{-1})	Cloudage
Jan	-17.3	71.7	174.6	2.3	4.6
Feb	-13.2	75.8	226.2	2.6	4.5
Mar	-0.7	49.1	330.0	3.6	4.4
Apr	9.4	44.9	327.9	4.4	4.1
May	15.7	50.4	352.3	4.1	4.5
Jun	20.8	62.7	329.8	3.3	4.7
Jul	23.8	70.5	319.3	3.6	4.6
Aug	22.2	70.7	328.5	3.3	4.5
Sep	16.9	59.8	321.8	3.2	4.3
Oct	7.1	50.8	264.8	4.2	4.4
Nov	-5.5	64.8	193.3	3.4	4.6
Dec	-14.0	70.1	172.0	2.6	4.7

Table S4. Monthly mean daytime meteorological parameters in Shanghai (hot summer and cold winter region).

Month	Ambient Temperature (˚C)	Relative Humidity	Solar Irradiation (W m^{-2})	Wind Speed (m s^{-1})	Cloudage
Jan	5.2	73.4	194.4	3.1	4.5
Feb	7.3	66.0	278.5	3.3	4.7
Mar	11.2	73.0	255.2	3.7	4.5
Apr	16.4	70.0	314.8	3.6	4.6
May	21.7	73.1	356.9	3.4	4.5
Jun	25.1	79.0	312.0	3.5	4.3
Jul	28.3	77.8	336.3	3.4	4.4
Aug	27.7	79.8	292.4	3.4	4.1
Sep	25.2	73.8	346.2	3.6	4.6
Oct	20.2	66.5	317.2	3.4	4.6
Nov	14.9	62.6	233.0	2.7	4.6
Dec	8.3	62.0	245.8	2.8	4.5

Table S5. Monthly mean daytime meteorological parameters in Kunming (temperate region).

Month	Ambient Temperature (°C)	Relative Humidity	Solar Irradiation (W m^{-2})	Wind Speed (m s^{-1})	Cloudage
Jan	11.8	51.4	368.0	1.9	4.5
Feb	13.3	51.9	409.4	2.8	4.5
Mar	17.2	41.9	462.2	3.6	4.5
Apr	20.0	45.6	482.8	3.2	4.6
May	20.4	61.6	365.8	2.7	4.6
Jun	21.6	69.1	338.7	2.1	4.4
Jul	21.0	76.6	310.8	1.6	4.5
Aug	20.9	75.1	317.1	1.2	4.6
Sep	20.2	72.6	351.2	1.7	4.4
Oct	18.2	70.6	319.5	1.6	4.3
Nov	13.5	63.8	303.0	1.9	4.5
Dec	10.4	61.0	308.0	1.7	4.6

Table S6. Monthly mean daytime meteorological parameters in Haikou (hot region).

Month	Ambient Temperature (°C)	Relative Humidity	Solar Irradiation (W m^{-2})	Wind Speed (m s^{-1})	Cloudage
Jan	19.0	81.0	202.9	2.9	4.6
Feb	19.9	84.1	222.8	2.3	4.4
Mar	22.4	80.9	251.9	2.9	4.5
Apr	26.5	80.7	321.5	2.4	4.6
May	29.0	76.1	366.7	2.5	4.6
Jun	30.0	76.7	356.7	2.2	4.7
Jul	30.5	73.4	417.1	2.4	4.5
Aug	29.8	76.7	393.9	2.0	4.6
Sep	28.5	80.6	358.6	2.7	4.3
Oct	27.1	76.2	294.1	2.3	4.4
Nov	22.9	74.3	243.5	3.0	4.1
Dec	20.4	75.2	207.3	2.5	4.4
